# Nurses’ perspectives on medication safety for Swedish community-dwelling older adults in primary care

**DOI:** 10.1080/02813432.2026.2690598

**Published:** 2026-06-27

**Authors:** Gabriella Caleres, Sarah Thelin, Elisabeth Persson, Veronica Milos Nymberg, Sara Modig

**Affiliations:** ^a^ Center for Primary Health Care Research, Department of Clinical Sciences Malmö, Lund University, Malmö, Sweden; ^b^ Department of Medicines Management and Informatics, Malmö, Sweden; ^c^ Primary Health Care Centers in Region Skåne, Sweden; ^d^ Office for Integrated Care, Malmö, Sweden

**Keywords:** Primary health care, medication safety, nurse’s role, health services for the aged, geriatric nursing

## Abstract

**Background:**

To improve care for community-dwelling older individuals, Swedish primary care has implemented nurse-led elderly care units targeting this group.

**Aim:**

To explore how primary care nurses working in elderly care units view their role regarding medication safety and gain their perspectives on the work model.

**Methods:**

This was a qualitative focus group study with 14 strategically sampled primary care nurses working at elderly care units. Data were collected through three focus groups and two individual interviews between September 2024 and June 2025, audio-recorded and transcribed verbatim. Content analysis was applied, and the person-centred nursing framework was used to interpret the results. Triangulation and reflexivity were pursued, and an audit trail was maintained to ensure trustworthiness.

**Results:**

Five categories emerged (1), diverse conditions for elderly care units (2), preventing and managing drug-related problems (3), bridging the gap – a vital function for medication safety (4), the comprehensive geriatric perspective, and (5) perceptions of the nurse’s role.

**Conclusion:**

This study found that nurses in elderly care units recognize their potentially significant role in medication safety. Their comprehensive geriatric perspective enables them to impede or intervene on drug-related problems, compensate for fragmented care and resolve issues as a central coordinator. However, the absence of guidelines leads to inconsistent and inequitable care, insights important for further development of the work model.

## Introduction

Drug-related problems (DRPs) are a significant cause of morbidity and healthcare utilization among older people [1]. As the population ages, the use of medications increases, leading to a higher risk of DRPs, which can include adverse drug reactions, suboptimal treatment, or misunderstandings regarding current prescriptions [2]. While older individuals have an increased vulnerability to DRPs due to pharmacokinetic and dynamic organ changes [3], many DRPs affecting elderly individuals are preventable [4]. Additionally, many vulnerable multimorbid individuals with extensive medication regimens are staying in ordinary housing [5]. Previous studies have shown that drug-related risk situations are common among community-dwelling older people [6–8].

For several years, primary care in Sweden has offered individuals over 75 years, living in ordinary housing, health assistance at elderly care units, an outpatient unit embedded within the local primary health care center. Unlike nursing homes, they serve independently-living older adults registered at the primary health care center. These units are adapted to the complex care needs of older patients with the goal of creating security for the patients and their relatives by providing comprehensive assessments including medication management and continuity of care. Typically, one or more nurses appointed to elderly care are primarily responsible for running the unit and are thus well positioned to identify and act on drug-related risk situations during the contacts with the patients. Although the Swedish Association of Local Authorities and Regions highlights dedicated elderly care units within primary care as a recommended model within person-centered and integrated care [9], the presence and organization of such elderly care units varies between different counties in Sweden and there is no uniform nationwide requirement or structure.

Elderly care units using comprehensive geriatric assessment have the potential to reduce hospital care utilization among vulnerable older adults [10]. However, studies evaluating drug-related activities in similar units are sparse [11]. A small local study showed that 72% of the patients affiliated with the elderly care unit used five or more medications, and that 44% of visits were made by individuals with diagnosed cognitive impairment [12]. This affects the individual’s ability to safely manage their drug treatment. More knowledge is needed on how elderly care units within primary health care can be optimized for the best medication safety for individuals affiliated with the clinics, especially from the nurses’ perspectives.

Responsibility to work towards increased safety in the individual’s medication management should be shared by all healthcare professions involved in the care of the older patient. Follow-up on medication changes, improved information transfer between healthcare providers, and empowering the elderly and their relatives with increased knowledge are important aspects. Multidisciplinary collaboration and communication between different health care professionals are essential for accomplishing this in an effective and efficient manner [13,14]. Nurses should be considered an important element in the health care team when evaluating and supporting medication management [13].

The aim of this qualitative focus group study was to explore how primary care nurses appointed in elderly care viewed their role regarding medication safety what experiences existed, and gain their perspectives on the work model.

## Materials and methods

### Study design

This was a qualitative study based on three focus group (FG) interviews and two additional individual interviews with participants who were unable to attend the focus groups. Focus groups are suitable to explore experiences and views in a group with shared experiences and knowledge such as health care professionals [15]. Data are generated through group interaction and shared reflections. However, it is important that the moderator facilitates the discussion so that all participants can voice their perspectives [16]. The reporting followed the Consolidated criteria for Reporting Qualitative research (COREQ) [17] (See Supplementary File 1).

### Study population and settings

The participants were nurses working at elderly care units within primary health care centers (PHCCs) in two different counties in Sweden. The respondents were contacted *via* email: in one county, all PHCC managers were asked to forward an invitation to the elderly care unit nurses at their centers; in the other county, the invitation was sent directly to all elderly care unit nurses *via* a mailing list. The recruitment was strategic to ensure both rural and urban PHCCs and participants with different work experience. Two nurses who had initially agreed to participate were unable to attend the interviews, resulting in a total of 14 participants. Recruitment and data collection were conducted parallel to the analysis and continued until additional interviews provided no further information in relation to the study’s aim. The assessment of sample size can also be considered within the principle of infor­mation power [18]. Given the strategic recruitment, the homogenous group of participants and the quality of the interviews, a high level of information power was acquired with a limited number of participants.

The first two focus groups consisted solely of participants from Skåne County, the third of a mixture of participants from Skåne and Östergötland counties. The two individual interviews were conducted with individuals from each county. All 14 participants were women, with a mean age of 48 years (range 31–63 years). Six had additional training in elderly care, two in cognitive diseases and two in palliative care. Four nurses had a postgraduate diploma in specialist nursing. Their allocated time at the elderly care unit varied from full-time to both open phone hours (with a specific phone number available for the elderly patients) and patient appointments in the PHCCs as well as home visits, to only providing two hours of open phone hours per day. Some of the nurses had a nursing colleague at their elderly care unit, whereas some worked unaccompanied. Both counties used electronic communication tools between all healthcare providers and municipalities for coordinated care and support planning for eligible patients.

### Data collection

Prior to the interviews, a letter was sent *via* e-mail to the participants, containing information about the background, purpose of the study, and some points to reflect on and contact details of the principal investigator in case of further questions. The interviews took place between September 2024 and June 2025. Written consent was collected from all participants. The focus group discussions were led by a moderator (ST) and accompanied by an observer (GC) taking notes, to ensure all information from the meetings was collected. All focus group sessions took place in person, with two of the groups having a single individual participating online. Two of the focus groups took place at one of the participating PHCC, one at a conference center. The individual interviews were conducted by ST and GC, respectively, one in person at the participants’ PHCC and one digitally. The focus group interview lasted from 1 h and 50 min to 2 h and 15 min, and the individual interviews between 40 and 60 min.

The interviews were based on a semi-structured interview guide exploring the participants’ perceptions of and experiences of how they contribute to medication safety (Supplementary File 2). Collaboration and communication with the responsible physician were covered, as well as patient education, in addition to open questions regarding current working methods. The interview guide was developed by the authors to reflect the aim of the study, with minor alterations being made after the first focus group. The interviews were audio recorded and transcribed verbatim. After proofreading, the recordings were destroyed. Transcripts were not returned to the participants for comments or participant checking.

### Analysis

A qualitative approach with content analysis according to Graneheim and Lundman was used, as it is a qualitative content analysis often used in healthcare research, designed to analyze such data in a systematic and transparent approach [19]. Accordingly, the interview transcripts were first checked for correctness against the audio recordings, then read through several times to become acquainted with the content. Meaning units were identified, condensed, and abstracted to codes by either author GC or ST. For validation, each interview was assessed by at least two authors, of whom one had been present during the interviews. Next, the codes were collectively compared and sorted into 21 subcategories and five categories by the entire research team. Agreement on the categorization was reached through discussion and reflection within the group. As the study sought to describe the participants lived experiences and perspectives, the analysis was limited to categories at a descriptive level [20]. An example of the analysis process is shown in Table 1. After the initial analysis, one of the individual interviews took place. Several authors read the additional interview, confirming the accuracy of the analysis as well as the saturation of the material.

**Table 1.** Examples of the analysis process.

Meaning unit	Code	Subcategory	Category
We help with everything, since we are the spider in the web. We know what happens in home care, at the hospital, at the pharmacy… everything, really. And that’s safe, I can arrange prescriptions from the GP or talk. It’s the best part, a sense of making a difference.	The elderly care unit nurse is the spider in the web with an overview of all stakeholders, which gives the patient a sense of safety and the nurse a sense of making a difference.	The coordinator	Perceptions of the nurse’s role
We work extremely hard [in the electronic communication tool] to ensure the information is right, we even got a hospital contact person as we were so meticulous regarding the timeliness of everything. We work hard for a safe discharge without medication discrepancies between the medical history and the discharge summary	Work hard to ensure correct and timely information in discharge documents to ensure a safe discharge without medication discrepancies	Lacking information transfer in care transitions	Bridging the gap - a vital function for medication safety

### Preunderstandings

All authors had experience with qualitative research methods as well as from working with elderly care in a primary care setting, four through practicing as general practitioners, and one from working as a registered nurse, with several of the authors having distinct tasks dedicated to elderly care. Moreover, several authors had an ongoing regional engagement for increased medication safety.

### Ethics

The Swedish Ethics Review Authority made an advisory statement (Registry number 2023-06885-01), issuing that an ethical approval was not mandatory. The participants received both written and oral information on the project, including the background and aim, their voluntary participation, anonymization of their quotes, and right to withdrawal. Informed consent was collected from all participants. Thus, recommendations from the Swedish Research Council were upheld [21]. Adherence to the Declaration of Helsinki principles was maintained [22]. Furthermore, no private or professional relationship existed between the researchers conducting the interview and the participants.

## Results

The analysis of the material indicates the overall importance of how viewing through geriatric lens forms the basis for working with medication safety.

The categories relating to the manifest content are outlined below under their corresponding sub-heading, with subcategories in *italics* built into the text (Table 2).

**Table 2.** Final categories and subcategories.

Diverse conditions for elderly care units	The comprehensive geriatric perspective	Perceptions of the nurse’s role	Bridging the gap – a vital function for medication safety	Preventing and managing DRPs
*Circumstantial differences* *Team communication* *Compensating for lack of continuity*	*Continuity and patient comfort* *Recognizing the complex needs of the elderly* *Accessibility* *Bridging the gap in the doctor-patient relationship* *Relationship with family members*	*The coordinator* *Self-perceived competence versus mandate to act* *Job satisfaction*	*Hospital discharge follow-up* *Lacking information transfer in care transitions* *Multidose drug dispensing (MDD) in care transitions* *Different caregivers and separate electronic medical records*	*Ensuring an accurate medication list* *Educating and supporting the patient for safe medication use* *Medication follow-up* *Identifying drug-related problems (DRPs)*

### Diverse conditions for elderly care units

This category entailed the consequences of the unclear prerequisites for the elderly care units leading to substantial differences in the organization and delivery of the service, as well as regarding how the health care team was structured and functioned.

*In light of the growing elderly population, I don’t understand why the investments in elderly care units haven’t been greater than vague guidelines and a limited financial compensation, do as you like more or less. It should be much clearer; all are individuals, and it should be individual, but the foundation ought to be the same for everyone. Otherwise, it’s not equitable healthcare. (FG 2)*

Hence, *the circumstantial differences* between PHCCs in leadership and time prioritization clearly affected their resource frame and therefore the possibility to provide equal care. Whereas some PHCC managers were perceived as committed to this working model, others were reluctant to improve it and provided unclear directions. Similarly, the elderly care unit was considered of utmost priority in certain PHCCs, with a shared perception that it reduced the workload for other health care professionals by directing these patients to a specialized service. In others, the time dedicated to the elderly care unit was extremely limited and constantly at risk of being cancelled in the event of staff shortages. Therefore, a wish for more time being allocated to this important task to make the most of its full potential was expressed.

These differences in set-up implied a greater need to exchange different experiences among the respondents, who felt this was enriching and stimulating. Whereas some elderly care units in addition performed other tasks such as cognitive assessments, others solely offered phone consultations during open phone hours. There was a lack of a common structure to identify eligible patients. In general, patients would contact the elderly care units *via* telephone or at the initiative of other caregivers or relatives. Some nurses would schedule appointments as needed, while others arranged annual visits. While some described using various assessment scales, others viewed such tools as too time-consuming to apply in practice. A tendency to become a catch-all for tasks that no one else had time for was also described.

The subcategory *team communication* concerned how well-functioning teamwork helped create a sense of safety and was essential for the nurse to work autonomously.

*We work as a team. The doctors ensure that the prescription is correct according to kidney function and other aspects, and we nurses take more care of compliance; that they take their medication, how do they take it. In this regard, we contribute significantly to medication safety, do they stop, why do they stop, are they worried or do they give up. (FG 3)*

Some were comfortable with communicating with the different patient-responsible general practitioners (GPs), while others preferred a designated elderly care unit physician. Among the various ways of communication, ward rounds were the most preferred. However, team communication was not always smooth. A degree of discontent regarding the GPs contribution was expressed, such as lack of updating medication lists, not acting on the nurses’ medical record notes and failure to initiate multidose drug dispensing. Furthermore, a lack of decisiveness by the GP and inability to adhere to the agreed care plan were reported to be frustrating. Some also expressed a sense of being sidelined when the GPs would not allow them to take over responsibilities.

On the other hand, GPs who knew their patients well were noted as comforting. But, due to the shortage of GPs, the nurses often had to *compensate for lack of physician continuity*. As one respondent explained:

*Earlier, there were more GPs, and the elderly care unit functioned differently. Now, we’ve had to rebuild given our situation. It’s fantastic to do that and that the nurse is still here and is the continuity at the core. (FG 1)*

Due to the shortage of GPs, nurses had to solve more tasks independently, relying on physicians mainly for consultations. In cases deemed appropriate, routine annual visits with a GP were replaced by appointments at the elderly care unit, thus making it especially important to document any interaction with the GP in the patient’s medical records. An especially heavy burden of responsibility was heightened when temporary physicians unfamiliar with the patients - were on staff, which made duties like medication follow-up more challenging.

### The comprehensive geriatric perspective

The comprehensive geriatric perspective covered various aspects of caring for elderly patients, including essentials such as taking an individual approach and providing *continuity and patient comfort*. Continuity was considered to make the patient feel more secure and confident in the nurse, encouraging them to seek care when needed and helping them to navigate, for example, challenging medication adjustments. The importance of being accessible to the patient and providing support through difficult periods was described:

*You almost take them by the hand and follow them through these rough weeks [after medication adjustments]. (FG 3)*

For patients with cognitive impairment, such support could be offered by frequent telephone follow-ups. Home visits were also described by some respondents to establish trust. The resulting enduring relationships were also rewarding for the nurse, and facilitated deeper conversations on, for example, quality of life.

As for patients, common shared traits were signs of cognitive decline and/or multimorbidity and often a recent change in medication. In addition to hospital discharge, these patient commonalities were typical causes for visiting the elderly care unit. Accordingly, a fundamental dimension of the nurses’ work concerned *recognizing the complex needs of the elderly*. This entailed utilizing their full range of knowledge and focusing on the frailest patients with the greatest need of care, ensuring that resources are used effectively. It also meant being a ‘perceptive detective’ and using a tailored approach, highlighting the critical importance of patient-centered care for this population:

*The most important thing is that equal healthcare is not to do the same for everyone, but to do what is necessary for everyone to reach the same goal, and it’s the same with medications. We need to inform in different ways; some by telephone, others face to face, you need to remind them, constantly print the medication lists, and review them critically. That’s the most important thing we do. (FG 2)*

This also involved everything from listening to life stories to initiating end-of-life conversations, as well as enabling and respecting well-informed patient decisions regarding medications.

Another way to embrace the preferences of this patient group and make them feel safe was to provide *accessibility*, such as by quick telephone accessibility with a person instead of an automated telephone voice menu. Another key responsibility was allowing patients to drop in for help without needing an appointment. This approach prioritized immediate needs, such as performing a medication reconciliation to avoid drug related problems. One respondent observed that patients felt that the elderly care unit had time for them, unlike the GP, which helped *bridge the gap in the doctor-patient relationship*. On occasion, patients stopped taking their medication due to side effects or lack of effect, often without anyone asking the reason. The nurse played an important role in medication follow-up, providing opportunities to clarify the GP’s prescription and address any questions that had emerged. Often, the patients were more forthcoming with the nurse regarding which medications they were actually taking. A lack of patient understanding about the reason for taking their medications was described as common, and most were unlikely to question the doctor’s decisions. Some patients would even agree to investigations or medications to please the GP. In these situations, the nurse’s ability to listen carefully and represent the patient’s interest became especially important.

Finally, the *relationship with the patient’s family members* was also a key aspect of the nurse’s broader care perspective. Family members often contacted the elderly care unit when the patient failed to handle their medication or something else malfunctioned. At times, discussions were tough when they indicated a need for extended help, which the patient refused to accept. Family members were recognized as a valuable source of support for patients. In return, nurses aimed to assist these family members as well, although limited time often made this challenging.

### Perceptions of the nurse’s role

In this category the benefits and challenges of being a nurse appointed to elderly care were described. Being a *coordinator* was described as an essential part of the role, facilitating good collaboration around the patient like a spider in the web. As such, identifying issues and organizing, for example, prescriptions from other caregivers were important tasks. In addition, nurses frequently communicated and collaborated with home care staff, especially when patients were discharged from hospitals with incorrect medication lists. When needed, these cases were forwarded to the GP for further action.

Another widely described aspect of the role was the perception of *self-perceived competence versus mandate to act*. Daring to question the GP’s prescription was viewed as important, but there was a wish for more feedback for learning purposes. At times, less experienced physicians would ask questions beyond the nurses’ competence and responsibilities, which led to a sense of insecurity when having to make decisions based on experience rather than knowledge. As one respondent said:

*It feels difficult when the doctors don’t know what to do and ask about things, I’m not qualified to take responsibility for. (FG 3)*

The common lack of physician geriatric competence and experience meant the nurses had to work more autonomously. Sometimes, this was limited by the lack of authority to make decisions regarding, for example, the patients’ medications. At times, they perceived that physicians were hesitant to deviate from treatment guidelines, despite significant side effects.

*If the patient is, for instance, experiencing dizziness, we typically ask whether it might be related to medications for blood pressure or heart failure. These issues are frequently discussed, but as we do not make the final decisions, we sometimes have to find alternative non-pharmacological solutions. (Individual interview 2)*

Working as an elderly care unit nurse was stimulating due to the constant learning process. To take overall responsibility and having long-term patient relationships was also very appreciated, as was the independence and opportunity to take personal initiative. A defining characteristic was the immense *job satisfaction* the role brought.

*There are endless possibilities and it’s the most fun job I’ve had in my career. It’s rewarding and the elderly are very grateful, and you get to work independently. (FG 1)*

The comprehensive perspective was also a contributing factor to the sense of occupational fulfilment, as it allowed the nurses to work with the whole person, addressing not only illness but also supporting and preserving overall health.

### Bridging the gap – a vital function for medication safety

A significant part of this category focused on the important task of *hospital discharge follow-up*. Many respondents followed up on medication changes and/or compliance as well as the patients’ wellbeing after discharge routinely or when needed. Most patients were identified through the electronic communication tool for admitted patients common to hospital, primary and municipal care.

*We identify admitted patients 75 years or older without home care nursing. The GP is provided with a checklist with multiple-choice options before hospital discharge follow-up from which they can decide on additional follow-up besides medication reconciliation which is always performed. (Individual interview 1)*

An important task was to check the medication list and make sure the patient had received all the necessary information and prescriptions, with the main purpose to prevent readmissions. However, not all elderly care units had a standard follow-up procedure. Some described follow-ups as the GP’s responsibility, and others as up to the patient. However, an increased risk of medication errors during this critical period was identified, especially in the case of delayed follow-up, which could also affect compliance and medication safety negatively.

A closely related area of discussion was the *lacking information transfer in care transitions*. The electronic communication system between different caregivers was described as an important tool in which discharge documents were transferred. Some respondents reported focusing greatly on the accuracy and timeliness of such discharge documents to ensure safe discharge without medication errors. As information transfer was otherwise worse due to different electronic medical records between different caregivers, this was a way of bridging this gap and facilitated follow-up.

Difficulties were also prominent regarding *Multidose drug dispensing (MDD) in care transitions*. There was consensus that initiating MDD (machine-dispensed disposable sachets in which medications are packaged according to the intended time of administration [23]), during hospital admission was beneficial. However, this seldom happened which caused irritation and delays, in turn leading to increased risk of medication errors. As one respondent explained:

*Some departments can’t start multidose drug dispensing, they want us to do it in primary care after discharge, which leads to delay. Moreover, we must await discharge medication orders/prescriptions since they may change at the end of the hospital stay. It would be more convenient if everybody could prescribe in the MDD system, so that everything was ready at discharge. (FG 1)*

Delays were also caused by GPs not wanting to prescribe other caregivers’ medications in the MDD system, which in itself is good for medication safety. Finally, delays were caused by GPs delegating the task of registration in the electronic MDD system to the nurse, which was also seen as time-consuming.

The final gap the nurses had to bridge was due to *different caregivers and separate electronic medical records*. All agreed that a common electronic medical record would be ideal. There was often confusion due to different medication lists from the pharmacy, primary care, the hospital and other caregivers. A special situation with risk of drug-related problems was when patients travelled to their home country and returned with new medications. This felt like ‘a ticking bomb’ according to one nurse. They considered the GP to be responsible for coordinating prescriptions from different caregivers, although the extent to which it worked varied.

### Preventing and managing drug-related problems

The final category entailed the strategies used to identify and prevent drug-related problems. First, *ensuring an accurate medication list* (i.e. medication reconciliation) was a highly prioritized task and viewed as one of the most important duties to aid medication safety.

*Basically, I just review the medications with the patient, ensure that the medication list matches what the patient is taking and should be taking, check how they are feeling, and confirm that they are taking them correctly or if there are any difficulties. In other words, all these things we are supposed to do. (FG 3)*

Incorrect lists were seen as a great obstacle and the importance of investigating thoroughly with questions to identify discrepancies was stressed. Such discrepancies were reported to the GP for medication list updates. However, this was not always the case after hospital discharge, even if the discharge documents were transferred. Working with medication reconciliations was described as time-consuming detective work across multiple sources, and it was rarely on allocated time. Nevertheless, taking time was considered crucial for medication safety. Again, a common list such as MDD was requested.

*Educating and supporting the patient for safe medication use* was a key element of preventing drug-related problems. As misunderstandings were common, clarity was essential for this age group. Many elderly patients didn’t know how or why they should take their medications, which is why follow-up and supplementary discussions were needed to allow a chance to ask questions.

*That we have reviewed the medications and then discussed* ***why*** *they are being taken and why they are important. (Individual interview 2)*

Patients with cognitive impairment might require repeated explanations regarding their medications – and this need was consistently met.

Nurses noted that unclear or inaccurate information about side effects could reduce patient compliance, while thorough explanations of medication changes improved it. They emphasized the importance of instructing patients when to pause medication and sometimes used interpreters to increase their understanding. The nurses also educated patients on using aids such as MDD, pill organizers or dispensers. A lot of effort was put into raising the patients’ awareness of the importance of an accurate medication list as well as on lists not matching current prescriptions.

As noted herein:

*I asked a patient to bring their medication list, and they came with the list from the pharmacy on remaining prescriptions which they used as their list, which is very common. There were medication duplicates in different doses, and they didn’t understand this wasn’t a proper medication list. (FG 1)*

This provided the nurse with a valuable chance to inform the patient of the importance of having a reliable and comprehensive medication list. Finally, the nurses provided support for patients with MDD and urgent medication changes, as this was often a bit complicated.

The nurses had an important role in *medication follow-up*, often on behalf of the GP, after adjustments of blood pressure or dementia medications. The nurses also participated in titration of medications and ensured adherence during dose changes, in addition to follow-up of their own prescriptions. A welcome side effect of being involved in such medication changes was increased competence.

Part of the job involved *identifying drug-related problems (DRPs)* to prevent unnecessary healthcare utilization. This meant a constant awareness of medications being the possible cause of any issues.

*To be observant and not assume the patient has simply deteriorated in their health but consider this might be due to medication. (FG 2)*

The nurses identified both expected as well as unwanted side effects such as gastrointestinal or dizziness, frequent urination, and ankle edema, sometimes complicated by a prescriber cascade. Extensive medication after cardiovascular events was common and often caused adverse effects. Polypharmacy was also often noted, as by this respondent:

*Our elderly patients shouldn’t have these long medication lists; it does more harm than good. (FG 2)*

Assessment tools were sometimes used, and lack of compliance noted. Common causes of DRPs were substitute medications and generic drugs.

Finally, facilitating interprofessional medication reviews was seen as another useful tool in preventing and managing DRPs, partly by providing patient support with information afterwards. Interprofessional medication reviews involve participation of a clinical pharmacist in addition to the patient-responsible physician. Some nurses reported a lack of medication reviews, and not all participated in those that took place. Often, they had more of a coordinating role or contributed by gathering facts or identifying suitable patients.

To summarize, the elderly care unit nurses provide continuity and support while adjusting to the individual patient’s unique prerequisites and needs to provide the best possible care. Serving as the central link, they adjust to varying circumstances while using their specific expertise for these patients. Simultaneously, they strive to improve medication safety by acting as intermediaries and diligently working to recognize and prevent potential medication errors by counteracting various organizational shortcomings and patient-related issues.

## Discussion

### Summary of main results

The aim of this qualitative focus group study was to explore how primary care nurses in elderly care units perceive their role in medication safety, their related professional experiences, and to gain their perspectives on the work model.

The nurses in our study play a potentially significant role in promoting medication safety in multiple ways, characterized by a comprehensive geriatric perspective in the work model. Despite large variations in working conditions in practice, nurses consistently make efforts to bridge the gaps caused by fragmented care and poor continuity, striving to ensure medication safety for elderly patients.

### Comparison with other studies

One of the theories used to explain factors of importance in nursing research is the person-centered nursing approach. The person-centered nursing framework consists of four domains [24]: 1) *Prerequisites*, including skills and abilities of the health professionals (such as competency, interpersonal skills and commitment to the job); 2) *The care environment* (effective staff relationships, power sharing and shared-decision making systems); 3) *Care processes* (such as working with patients’ beliefs and providing a holistic approach); 4) *Person-centered outcomes* (such as satisfaction and involvement with care). As the person-centered nursing approach uses different domains, which are common for Swedish nurses working with elderly patients in primary care, we have chosen to theoretically contextualise our findings through the lens of this theory.

As to *the nurses’ prerequisites*, the participants in the current study often worked very independently, showed great commitment and gained significant reward from their work. Such satisfaction has also been noted for nurses working with elderly patients in diverse settings [25,26], suggesting it’s a main shared motivating factor. However, such autonomy could also entail limited opportunities for consultation or support. An essential part of the nurse’s role is being the spider in the web, coordinating care across health care providers, including medication management. This is in line with other studies also identifying the primary care nurse as a potential health professional key player in preventing and reducing medication discrepancies in care transitions [27,28]. As stated in one narrative review, nurses hold a pivotal role in identifying patients at risk for DRPs and serve as communication links between caregivers as well as with patients and their relatives [28].

However, this requires that they are given the optimal conditions to make full use of their skills. A Swedish study emphasizes the importance of good working conditions and collaboration as key factors influencing both the process and quality of medication evaluation for older adults [14]. Given this, there is reason for concern that the nurses in our study apparently work in contexts with significantly varying conditions. These variations could potentially limit the nurses’ ability to work at their full capacity in preventing DRPs. However, some consistent aspects appeared to provide support and help maintain effective care. Despite notable differences in organizational structures and working conditions, *the care environment,* defined as the context in which care is delivered, demonstrated shared characteristics, particularly the significance of team collaboration in supporting nurses’ autonomous clinical role. Teamwork is vital in the care of multimorbid elderly patients [29]. A recent systematic review suggested that interprofessional collaboration in primary care might improve health outcomes in this group although the findings were mixed [30]. Nevertheless, care quality was perceived as higher in collaborative settings, and some studies reported reduced health care utilization [30]. A common issue is that the interventions are rarely fully comparable. This also resonates with the different designs of the elderly care units – highlighting the need for standardized structures to ensure equitable care. In a qualitative study, other healthcare professionals emphasized the expertise and care coordination skills of specialist gerontology nurses in primary care [15], underscoring their role as a central team liaison. They were also considered particularly important for post-discharge follow-up, and valued for saving time for other professionals, as supported by other studies [15,31]. In line with this, nurses in our study sometimes had to compensate for a lack of physician continuity. Staff shortages are known barriers to teamwork, and a thriving care environment requires a balanced mix of professionals, each contributing their specific expertise [31]. A recent Swedish report stressed that the ongoing transition to person-centered and integrated care was hindered by unclear control strategies and insufficient resource expansion, particularly the critical shortage of GPs [32]. Hence, nurses should be viewed as collaborators rather than substitutes for GP shortages, in achieving the best possible care. While collaboration is valued [25], friction can occur, such as when GPs fail to update medication lists or act on requests for initiating multidose drug dispensing. At the same time, nurses are not authorized to make medication decisions, despite sometimes having greater experience. This may also place them in a challenging position when asked for clinical advice. Concurrently, to query the GP’s prescription was viewed as important and may support medication safety. There was also a wish for more feedback for learning purposes, which could be of value as communication and sharing is crucial for teamwork and interprofessional collaboration [31]. The importance of continuous learning and its positive effect on patient outcome is previously noted [25], and may, in addition, be an important motivating factor in terms of attracting nurses to a workplace.

Elderly patients with a high risk of hospital admission have higher rates of both mortality and hospitalization [33]. In a Swedish study, outpatient care based on comprehensive geriatric assessment led to prolonged survival and reduced hospitalization time [34]. This highlights the importance of identifying suitable patients for preventive measures, such as optimizing medication treatment for common hospital admission reasons, including heart disease and falls [33]. Meanwhile, a person-centered approach is of vital importance in *the care process* when addressing health issues in elderly patients and involves adopting a more holistic perspective [25]. Addressing complex needs with an individually tailored approach with medication management as an integral component was a core aspect of the practice for the nurses participating in our study. Being accessible, establishing trust, and providing continuity allowed for open communication, as previously noted [25,29]. As a result, the nurse is ideally placed to identify discrepancies and provide medication education in a way that is both effective and appreciated by both patients and their family members. Another key element of being an ambassador of medication safety was bridging the gaps, including compensating for a lack of medication information, which is a common issue in care transitions [28,35]. Also, ensuring an accurate medication list was regarded as an essential responsibility. Accordingly, nurses’ role in ensuring reliable medication information is pivotal to medication safety and should be a clearly prioritized task in elderly care units, most notably in care transitions.

A desirable *person-centered outcome* is to ensure a positive experience for both elderly patients and their families [25]. In our study, providing nurses with more time seemed to enhance patients’ perception of care quality. Another important aspect for patient satisfaction, medication safety and adherence was the nurse’s educating and supporting role. For example, counteracting confusion between brand and generic drug names and clarifying incomplete instructions [28], may enhance patients’ understanding and overall health literacy [15], Additionally, better medication quality has been associated with improved quality of life for elderly patients with polypharmacy [36].

### Strengths and limitations

Utilizing a theoretical framework to guide the interpretation of our results represents a strength of the study. Additionally, including direct quotes from the transcripts in the manuscript enhanced the credibility and trustworthiness of our results. In terms of reflexivity, all involved researchers had experience working in elderly care, which enriched the focus group discussions and interviews by providing valuable insights. However, this background may also reflect preconceived assumptions about the nature of the nurses’ work with the risk of confirmation bias. To address this, all researchers participated in the data analysis process. Triangulation by bringing together different perspectives stemming from different work experiences as well as different professions (GPs and nurses) helped minimize the risk of potential researcher bias and allowed for a richer and more nuanced interpretation of the findings.

Focus group discussions were chosen as an effective method to explore experiences and perspectives [16]. In addition, the individual interviews added further depth to the data. Both focus group discussions and interviews are qualitative methods, and were both conducted using the same interview guide. Whereas focus groups allow for more interaction between the participants, interviews tend to be more in-depth. These different perspectives and levels of depth constitute a strength and helped provide a rich and varied material. However, the combination of methods should be considered during the analysis to ensure a balanced perspective, and both interview and focus group data are reflected across all categories. Being a qualitative approach, there is a risk of interview bias, with the researcher unintentionally influencing the participants’ responses as well as participant bias, with the participants responding to please the interviewer. This was mitigated by using open-ended questions, ensuring a confidential and safe environment and the presence of a researcher as an observer, who was able to detect and correct potential bias or leading tendencies. Another limitation with the study design is the risk of selection bias. This was reduced by strategic sampling of nurses from PHCCs in both rural and urban areas. As for transferability, the findings are applicable to nurses working in similar settings. Nonetheless, as discussed previously, similar experiences have been reported in countries outside Sweden as well. Conducting the study across two different regions is a strength, as it reflects similar experiences, but not identical contexts.

### Future research

Further exploration of the nurses’ professional competence, knowledge and beliefs related to medicines may provide valuable insights. Future research should focus on developing and evaluating a uniform work model that supports nurses in safeguarding medication management for older patients. In addition, such knowledge may serve as a basis for further structured discussions with key stakeholders to facilitate implementation and organizational support.

### Conclusion

In conclusion, nurses acknowledge their vital role in ensuring medication safety within elderly care units, ideally working in close collaboration with GPs. Their regular contact and established relationships with patients place them in a strong position to detect DRPs including non-compliance, and to help resolve these issues by serving as the spiders in the web. Still, to fully realize the potential benefits of elderly care units for medication safety, it is essential to provide sufficient resources, including adequate staffing. Additionally, implementing standardized procedures and clearly defined prerequisites/roles is necessary to ensure consistent and equitable care.

This study adds important knowledge about the nurses’ work in elderly care units in Swedish primary care. These insights can support policymakers with evidence in making informed decisions about future healthcare strategies.

## Supplementary Material

Supplementary file 2 interview guide.docx

Supplementary file 1 COREQ.docx
