# Supplementary material for: Nurses’ perspectives on medication safety for Swedish community-dwelling older adults in primary care
Source: Scand J Prim Health Care. 2026 Jun 27;44(1):2690598. doi: 10.1080/02813432.2026.2690598 (PMC13312832; doi:10.1080/02813432.2026.2690598)
Supplement: Supplementary file 2 interview guide.docx [file IPRI_A_2690598_SM8178.docx]

**Supplementary File 2. Semi-structured interview guide**

Participant background information:

Name, additional training, years working in the elderly care unit, proportion of employment dedicated to the elderly care unit.

Which Primary Health Care Center (PHCC), duration of the elderly care unit at the PHCC, number of nurses working at the elderly care unit?

Overarching theme:

Tell us about your experiences and perceptions of working at the elderly care unit regarding medication-related issues for enrolled patients. Feel free to give examples from your practice.

Checklist:

1. Identifying different medication-related problems/risk situations:
   1. Example of a handled medication-related issue/problem. How did you manage it?
   2. The most commonly identified medication-related problem? Cite an example.
   3. Ways to inform/educate the patient regarding medications.
2. Participating in medication reviews:
   1. Are multidisciplinary medication reviews performed for patients without home care, and, if so, what is your role?
   2. Is Phase 20 used in preparation for multidisciplinary and/or standard medication reviews? If so, what is your role?
   3. Do you hold a coordinating or other professional function in relation to multidisciplinary medication reviews for patients receiving home care?
3. Follow-up of medication changes in primary care:
   1. Are you involved in the follow-up of medication changes for patients enrolled in the elderly care unit?
   2. Are there instances in which patients are enrolled at the elderly care unit solely for the purpose of follow-up on medication changes? What are the underlying reasons for such referrals?
   3. Are there structured procedures regarding your follow-up of medication changes and ensuring adherence to the revised prescriptions?
   4. Do you hold a coordinating or other professional role concerning medication lists for patients with multiple prescribers, including both the PHCC and outpatient units?
4. Hospital discharge follow-up:
   1. Are you involved in the review of patients’ medication lists in connection with hospital discharge?
   2. How do you work to minimize the risk of medication list discrepancies following hospital discharge?
   3. Are you involved in the follow-up of medication changes made during hospital stays? If so, in what way?
5. Collaboration with the patient-responsible General Practitioner (GP):
   1. Are there designated elderly care unit physicians within your PHCC?
   2. Is there a fixed time allocated for you and the designated physician to discuss medication-related or other issues associated with the elderly care unit?
   3. How do you communicate with other GPs regarding medication-related issues for their patients who are enrolled in the elderly care unit?
   4. How is a patient enrolled in the elderly care unit? Is there a template/checklist, or other tool to facilitate this? Which professional groups contact you when they identify the need for a patient to be enrolled?
6. Participation in cognitive assessments:
   1. Are you involved in conducting cognitive assessments at your PHCC?
   2. Do you collaborate with physicians to ensure that patients with cognitive disorders receive the appropriate medication based on their specified diagnosis? Do you perform the medication follow-up?
   3. Ways to support patients with cognitive impairment.
7. Collaboration with home care staff:
   1. Do you have regular meetings with the dementia nurse or other home care staff regarding patients who are beginning to have difficulties managing their medications?
   2. How is a patient transferred from the elderly care unit to home care when assistance with medication management is needed – by referral, telephone, via a physician, or do the patient/relatives contact home healthcare themselves?
   3. Are you involved in the communication process between physicians and home healthcare nurses in any way when a patient’s medication list is inaccurate, for example, after hospital discharge? If so, how?
8. Patient safety:
   1. How do you perceive your role in patient safety?
   2. What barriers do you encounter to an effective/patient-safe way of working?
   3. Wishes and needs for future initiatives to facilitate your work?

Conclusion:

1. What do you consider to be the most important actions you take to ensure medication safety?
2. Summary of the discussion
3. Anything missed or any points you feel have not been addressed?
